# Higher cytotoxic activities of CD8^+^ T cells and natural killer cells from peripheral blood of early diagnosed lung cancer patients

**DOI:** 10.1186/s12865-023-00553-4

**Published:** 2023-08-14

**Authors:** Mohamed Labib Salem, Ismail Atia, Nehal M. Elmashad

**Affiliations:** 1 Zoology Department, Faculty of Science, Tanta University, Tanta, 31527 Egypt; 2 Center of Excellence in Cancer Research, New Tanta University Teaching Hospital, Tanta, Egypt; 3 Zoology Department, Faculty of Science, Al-Azhar University, Assuit, Egypt; 4 Oncology Department, Faculty of Medicine, Tanta University, Tanta, Egypt

**Keywords:** Cytotoxic T cells, Cytokines, Granzyme B, Immune cells, Lung Cancer, Natural killer cells

## Abstract

**Introduction:**

Cytotoxic (CD8+) and natural killer (NK) cells play critical roles in anti-tumor immunity. Dysfunction in these cells is considered as one of the extrinsic mechanisms for tumor relapse.

**Aim:**

We aimed in this study to assess cytotoxic activities of CD8 + T and NK cells in the peripheral blood from lung cancer patients before and after induction of chemotherapy.

**Subjects and methods:**

Healthy (n = 5) volunteers and lung cancer patients (n = 15:5 before, 5 during, and 5 after induction of chemotherapy) were recruited. Flow cytometry was used to analyze the numbers of CD8 + T cells, NK and CD56^+^T cells and their intracellular expression of granzyme B (GzB) in fresh peripheral blood mononuclear cells (PBMCs) and after 72 h of their culture in vitro and stimulation with 5 µg/ml Concanavalin A (Con A) and 50ng/ml IL-2). In addition, the plasma levels of inflammatory cytokines were measured using luminex.

**Results:**

After culture, significant increases in the number of GzB expressing cells gated on CD3+, CD4+, CD8 + and NKCD8 + T cells in the PBMCs from lung cancer patients before induction of chemotherapy as compared to control individuals as well as patients during and after induction of chemotherapy. Serum levels of IL-1 and CXCL8 in patients before induction of chemotherapy showed 37- and 40-fold increases, respectively, as compared to control individuals. Both GzB expression and cytokines levels in patients during and after chemotherapy were similar.

**Conclusion:**

Polyclonal stimulation of PBMCs can restore the cytolytic activities of cytotoxic CD8 and NK cells from lung cancer patients even after chemotherapy.

## Introduction

The progression of cancer is known to be linked with an overall weakened antitumor immunity [1]. Therefore, novel approaches for its treatment have been developed with the key objective of inhibiting this attenuated immune mechanism [2]. Tremendous progress has been made to unveil the mechanism of the immune cell defense against cancer cells, presenting new and promising therapies based on new immunotherapy [3], in particular immune checkpoint inhibitors, antigen-specific vaccines, and adoptive T cell therapy [4], with the main goal to augment immune responses in targeting cancer cells [5]. These immunotherapies can potentially provide long lasting cancer remission without causing the serious side effects caused by radiation and chemotherapy, however low doses of chemotherapy may have an important role in changing the tumor area from cold to hot area which is necessary for the immunotherapy [6, 7]. In addition, they hold great promise in improving the prognostic outcomes of lung cancer patients, overcoming the hurdle of the poor overall 5-year survival rate barely ranging between 5 and 15% [8].

Lung cancer cells can induce an immune response that may associate with cytolytic immune cells including cytotoxic T cells (CD8^+^) and natural killer cells (NK) [9]. These cells are able to attack cancer cells via the release of the cytolytic content of their granules such as perforin (Pr), granzymes (Gzs), and interferon-gamma (IFN-γ) into the cytosol of targeted cells, leading to lysis of target cancer cells [10]. Among Gzs, GzB poses the major constituent excreted by CD8^+^ T cells and NK cells; it holds the strongest apoptotic activity of all serine proteases [11]. Therefore, the ability of CD8^+^ T cells to secrete high levels of these cytolytic mediators plays a vital role in anti-tumor effects.

Previous studies have demonstrated that lung cancer is associated with a locally decreased expression of GzB, Pr and IFN-γ by tumor-infiltrating CD8^+^ T cells, NK cells and NK T cells in the tumor microenvironment [12]. These effects are mediated by prostaglandin E2 (PGE2), transforming growth factor (TGF)-β) which promote tumor cell proliferation, anti-apoptotic properties, angiogenesis, and chemotherapeutic resistance [13, 14]. These immune alterations within the tumor microenvironment impair the functionality of cytotoxic CD8 + T cells and NK cells, resulting in reduction in the effectiveness of anti-tumor immunity [15].

In our recent study on newly diagnosed lung cancer patients, we have reported decreases of the numbers of cytotoxic CD8 + T cells and NK cells in association with reduction in their expression of GzB [16]. In this study, we aimed to compare the functionality of CD4^+^, CD8^+^, NK^+^, NKCD4^+^ and NKCD8^+^ T cell in the PBMCs of lung cancer patients before, during, and after induction of chemotherapy by analyzing their expressions of GzB. We also aimed to address whether if any alterations in the functionality of these cells is linked to the clinical response of the patients.

## Subjects and methods

In this study, 5 healthy individuals and 15 patients of lung cancer (13 male and 2 females) before, during and after induction of chemotherapy were included. Patients were in the median age of 55.52 ± 4.81 years with a long history of smoking (30 to 40 years). Patient’s demographics have been described in Table 1. Patients were diagnosed with NSCLC (poorly differentiated Adenocarcinoma) Table 2. Clinical data were correlated with the functionality of T, NK (CD56^+^) and NK T (CD3^+^CD56^+^) cells (Table 3).

**Table 1:** Demographic data in patient and control groups

Parameter	Control		Patients					
			Before%		During%		After%	
**Sex%**								
Male Female	80.0 20.0		90.0 10.0		90.0 10.0		100 0	
**Age**								
Min. – Max. Mean ± SD.	33.0–40.0 36.32 ± 2.43		35.0–75.0 55.52 ± 4.81					
Smoker%			**> 0.05**		**> 0.05**		**> 0.05**	
Nil Ve- Ve+	100.0		0.0		0.0		0.0	
	0.0		10.0		10.0		0.0	
	0.0		90.0		90.0		100.0	
			< 0.002*		< 0.002*		< 0.001*	
**p**_**Control**_			**0.034** ^*^		**0.027** ^*^		**0.012** ^*^	
**Performance**	No.	%	No.	%	No.	%	No.	%
0	0	0.0	1	20.0	0	0.0	5	100.0
1	0	0.0	2	40.0	3	60.0	0	0.0
2	0	0.0	2	40.0	0	0.0	0	0.0
3	0	0.0	0	0.0	2	40.0	0	0.0
Healthy	5	100.0	0	0.0	0	0.0	0	0.0
**p**_**Control**_			^**FE**^ **p=0.007** ^*^		^**FE**^ **p=0.007** ^*^		^**FE**^ **p=0.008** ^*^	

**Table 2:** Treatment with clinical parameters in patient

Parameter	Patients					
Treatment	Before		During		After	
Number of indicated cycles	No.	%	No.	%	No.	%
Nil	5	100.0	0	0.0	0	0.0
Positive	0	0.0	5	100.0	5	100.0
2 Cycle	-	-	0	0.0	0	0.0
3 Cycle	-	-	3	100.0	1	20.0
4 Cycle	-	-	0	0.0	2	40.0
5 Cycle	-	-	0	0.0	1	20.0
6 Cycle	-	-	0	0.0	1	20.0

**Table 3:** Different hematological and clinical parameters for patient and control groups

Parameter	Control	Patients		
		Before%	During%	After%
**Hb (g/dl)**				
Min. – Max.	11.3–16.3	11.1–14.8	8.75–11.0	8.4–12.4
Mean ± SD.	14.55 ± 2.43	12.72 ± 1.55	10.15 ± 1.18	10.3 ± 1.0
**p**_**Control**_		**0.732**	**0.824**	**0.643**
**WBCs/mm3**				
Min. – Max.	4750.0–10750.0	4.000–14750.0	3420.0–5500.0	2020.0–3700.0
Mean ± SD.	7254 ± 2245.0	9062.0 ± 2349.0	3725.0 ± 1724.0	2550.0 ± 450.9
**p**_**Control**_		**< 0.05** ^*^	**< 0.05** ^*^	**< 0.01** ^*^
**PL/mm3**				
Min. – Max. Mean ± SD.	150000.0–420000.0 2,770,833 ± 8730.9	120000.0–138000.0 128000.0 ± 1073.0	33,000–52,000 35,000 ± 1439	35400.0–38000.0 26,625 ± 29,217
**p**_**Control**_		**0.034** ^*^	**0.027** ^*^	**0.012** ^*^
**PBMCs / ml after Ficol**				
Min. – Max.	1.27–1.37	0.53–1.50	0.47–0.65	0.48–2.50
Mean ± SD.	1.31 ± 0.05	1.01 ± 0.44	0.60 ± 0.08	1.17 ± 0.85
**p**_**Control**_		**0.310**	**0.008** ^*^	**0.690**
**Lymph %**	31.5	69.36	54.95	49.85
**Lymph (10** ^**3**^ **)**	2.6	2.84	1.48	0.77
**Mono %**	3	8.4	7.8	6.025
**Mono (10** ^**3**^ **)**	0.255	0.344	0.210	0.093
**Gran %**	60	22.64	39.025	42.313
**Gran (10** ^**3**^ **)**	5.1	0.92	1.05	0.65

FE: **Fisher Exact** MC: **Monte Carlo**

p_Control_: p value for comparing between control and each other group

p_1_: p value for comparing between **during** and **after**

*: Statistically significant at p ≤ 0.05

Patients were recruited to Oncology Department, Tanta University Hospital. Blood samples were obtained from patients who given informed consent under a protocol approved by the Faculty of Medicine, Tanta University Ethical Committee Review Board who confirmed that all experiments were performed in accordance with relevant guidelines and regulations of Tanta University, Egypt. Patients were diagnosed according to The World Health Organization (WHO) criteria based on TNM classification [17]. Classification of subjects was performed after the detection of inclusion and exclusion criteria before, during and after induction of chemotherapy. Selection of patients with a clear history of treatment with broad spectrum antibiotics was carefully considered especially before chemotherapy treatment since the effect of antibiotics can affect the normal lymphocyte activation as ahs been proved when using immune activation in-vitro or after applying immunotherapy [18].

### Chemical and reagents

Lymphocyte separation medium (Ficoll paqué ™) was purchased from Lonza (Basel, Switzerland). RPMI 1640 was purchased from Biochrom (Berlin, Germany) human serum was purchased from Bio Whittaker ( Walkersville, MD), L-glutamine was purchased from Life Technologies (Paisley, Scotland), penicillin, streptomycin, Concanavalin A (Con A) and Brefeldin A was purchased from Sigma (Sigma Chemical Co., Munich, Germany), Interleukin-2 (IL-2) were obtained from R&D Systems (Minneapolis, Minnesota, USA). Phosphate buffer saline (PBS) was obtained from (Verviers, Belgium). CD3 (perCP.Cy5•5), CD3 (APC) clone (RXB-14/IG), CD4 (Allophycocyanin APC-A), clone ( SK3) CD56 (FITC), granzyme B (GzB) Phycoerythrin (PE) clone(RXB-14/IG9), FACS Perm were purchased from (BD Biosciences (BD), San Jose, CA, USA). Sheath Fluid was purchased from (Luminex Corp, Austin, TX, USA).

### Preparation of PBMCs

For plasma separation, blood samples were collected in K_2_EDTA tubes. PBMCs were separated with Ficoll paqué™ method using break off centrifugation. The cells were collected and washed using cold PBS and then counted using hymocytometer to assess the cell viability by trypan blue assay of PBMCs before in-vitro culture [19].

### Surface and intracellular staining of PBMCs for flow cytometry

After PBMCs separation using Ficoll, 200,000 cells were separated immediately after counting for surface staining of anti-CD3 (perCP.Cy5.5), anti-CD4 (APC-A) and anti- CD56 (FITC) and intracellular for GzB by using anti-GzB (PE) [20].

### Culture of PBMCs

PBMCs (1 × 10^6^ cells/ml) were cultured incomplete RPMI 1640 medium supplemented with 2% human serum FPS, 2 ml M L-glutamine, 50 units/ml penicillin, and 50 ng/ml streptomycin in the presence of 5 µg/ml concanavalin A (Con A) and 50ng/ml interleukin-2 (IL-2). The cultured cells were then incubated for 72 h in CO_2_ incubator at 37 °C in an atmospheric pressure of 5% CO_2_. Brefeldin A was added to the cultured cells 3 h before harvesting the cells for intracellular assessment of cytokines [21].

### Measurement of plasma cytokines and chemokines

Luminex 200 xMAP Technology (Luminex Corp, Austin, TX) was used to measure plasma levels of C-C motif ligand2 (CCL2), C-C motif ligand4 (CCL4), C-C motif chemokine 11 (CCL11), C-X-C motif chemokine ligand8(CXCL8), C-X-C motif chemokine ligand 10 (CXCL10), IL-1, IL-5 and IL-6 in plasma. Briefly, 50 µL plasma was diluted to two folds then incubated with specific label beads (Molecular Probes; Life Technologies, Carlsbad, CA) coated with antibodies. After several washes and incubation periods, the beads were detected with Luminex system (Luminex Corp, Austin, Tex, USA). Data were obtained and calculated using a 5-parametric curve fit using xPONENT®, version 4.03 in a blinded fashion with measurement performed with the FlexMAP3D system (Luminex Corp, Austin, Tex, USA) [22].

### Statistical analysis

Data were analyzed using SPSS software version 22 and Mann–Whitney or one-way analysis of variance (a nova) with post-hoc for non-parametric analyses. Correlations were performed using Spearman’s rank test. Analyses were performed using SPSS software. p values < 0·05 were considered significant.

## Results

### Reduction in the numbers of T, NK and BK T cell populations in fresh PBMCS of lung cancer patients

**Fig. 1:**
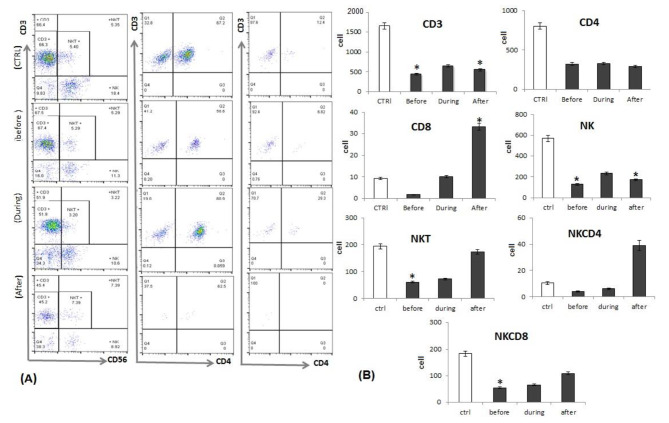
**(A)** Representative Flowcytometry analysis of CD3, CD8, CD4, NK, NKCD4 and NKCD8 cells from healthy control, lung cancer patients before, during and after induction of chemotherapy (before culture). **(B)** count of CD3, CD8, CD4, NK, NKCD4 and NKCD8 cells from healthy control (white bars), lung cancer patients before, during and after induction of chemotherapy (black bars) before culture

Using flow cytometry, we first analyzed the numbers of the total T cells (CD3^+^), T helper cells (CD3^+^CD4^+^), cytotoxic T cells (CD3^+^CD8^+^), NK cells (CD56^+^CD3^−^), NK T cells (CD56^+^CD3^+^), NK CD4^+^ T cell subsets (CD56^+^ CD3^+^CD4^+^), and NK CD8^+^ T cell subset (CD56^+^CD3^+^ CD8^+^) populations in the fresh blood samples from lung cancer patients (before, during and after) induction of chemotherapy as compared to control subjects. We found that, number of total T cells, T helper cells, NK cells and NK CD8^+^ T cell subset populations were decreased significantly in lung cancer patients (before, during and after)induction of chemotherapy as compared to control subjects, while cytotoxic T cells and NK CD4^+^ T cell subsets were decreased significantly in lung cancer patients before and during induction of chemotherapy and noted to be increased significantly in lung cancer patients after induction of chemotherapy as compared to control subjects (Fig. 1**).**

### Reduction in the expression levels of GzB by T, NK and CD56^+^T cells in fresh PBMCs of lung cancer patients

**Fig. 2:**
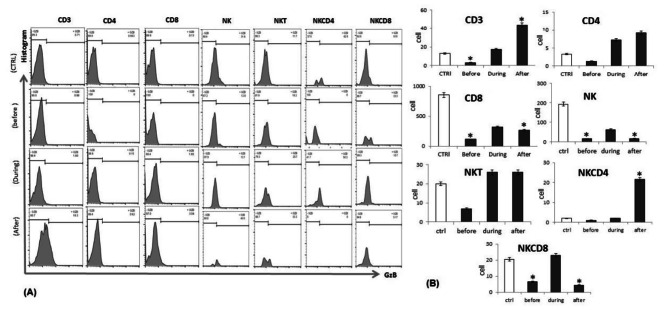
**(A)** Representative Flowcytometry analysis of GzB expression CD3, CD8, CD4, NK, NKCD4 and NKCD8 cells from healthy control, lung cancer patients before, during and after induction of chemotherapy (before culture). **(B)** Levels of Intracellular GZB expression on CD3, CD8, CD4, NK, NKCD4 and NKCD8 cells from healthy control (white bars), lung cancer patients before, during and after induction of chemotherapy (black bars) before culture

After we analyzed the numbers of immune cells above, we then used flow cytometry to measure the intracellular expression of GzB in the cytolytic immune cells (Fig. 2**)**, including cytotoxic CD8^+^ T, CD4^+^ helper T cells, NK, CD56^+^T, CD4^+^ NK T and CD8^+^ NK T cells in the same fresh blood samples in which we analyzed the cell numbers and phenotypes above. We found significant decreases in the expression of GzB in all analyzed cells populations in patients before induction of chemotherapy. The expression of GzB increased significantly in T helper cells, NK T cells and NK CD8^+^ T cell subset populations in patients during induction of chemotherapy as well as in CD4^+^ NK cells after induction of chemotherapy. Interestingly, however, expression of GzB also decreased significantly in cytotoxic T cells, NK cells and CD4^+^ NK cell subsets in patients during induction of chemotherapy as compared to healthy subjects (Fig. 2**)**.

### Lung cancer dysregulates the plasma levels of cytokines and chemokines

**Fig. 3:**
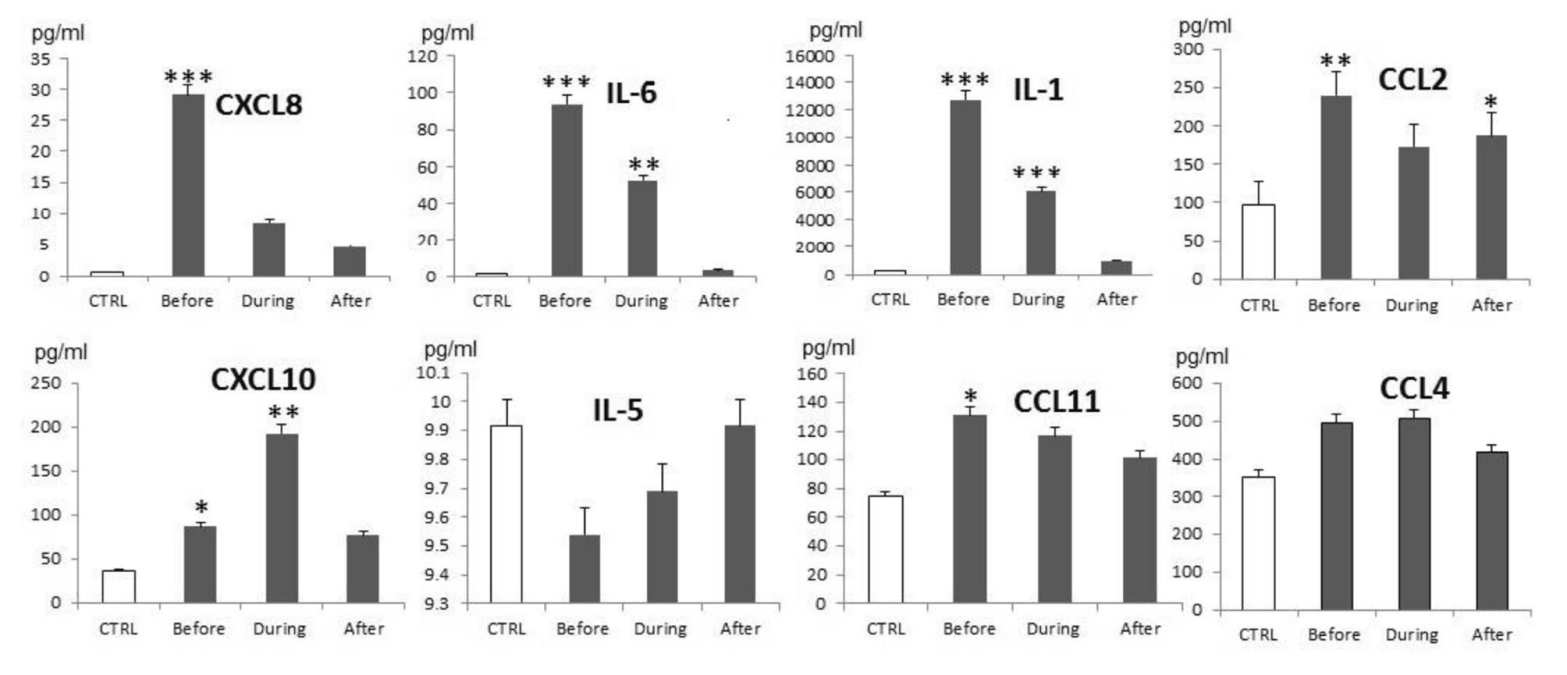
Statistical analysis of the expression of CXCL8, IL-6, IL-1, CCL2, CXCL10, IL-5, CCL11 and CCL4 in the plasma of lung cancer patients before, during and after induction of chemotherapy (black bars), compared to healthy control (white bars). Data were analyzed using SPSS software and Mann–Whitney or one-way analysis of variance (a nova) with post-hoc for non-parametric analyses, P-values < 0•05 were considered significant (*,**and *** p ≤ 0.05, p ≤ 0.01 and, p ≤ 0.001.(

After we analyzed the impact of cancer and chemotherapy on the numbers of lymphocyte subsets **(**Fig. 1**)** and their expression of the cytolytic marker GzB **(**Fig. 2**)**, we then wanted to link these effects on the inflammatory milieu represented by proinflammatory and inflammatory cytokines and chemokines. A shown in Fig. 3, patients before induction of chemotherapy showed significant increases in the levels of CXCL8, IL-6, IL-1, CCL-2, CXCL10 and CCL11 as compared to healthy controls. Patients during induction of chemotherapy showed significant increases in the levels of IL-6, IL-1 and CXCL10 as compared to healthy individuals. Interestingly, lung cancer patients after induction of chemotherapy showed no significant change expect for increases in the level of CCL-2.

### Lung cancer patients express low levels of GzB expression by in vitro activated T, NK and CD56^+^T cells in PBMCs

**Fig. 4:**
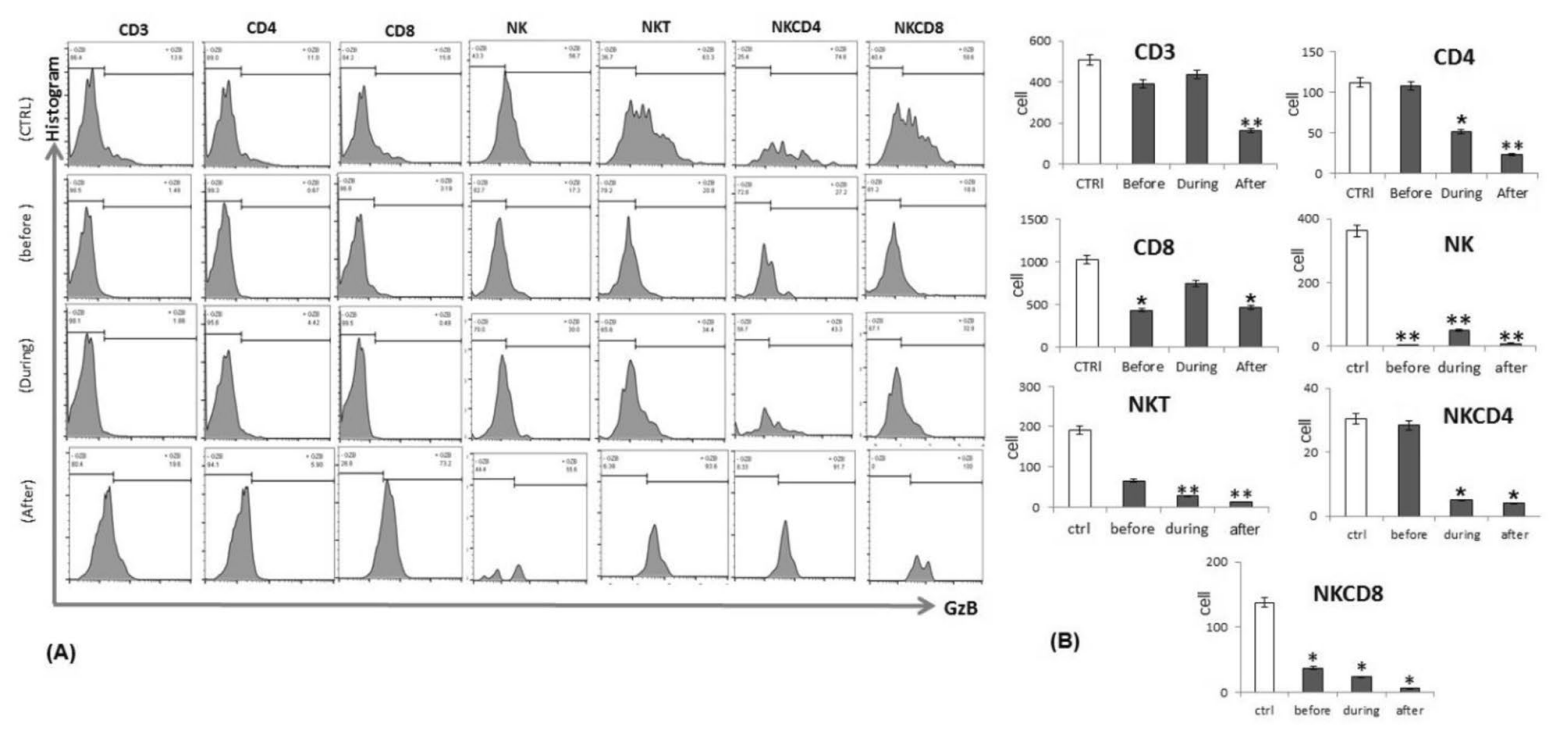
**(A)** Representative histogram of Flowcytometry analysis of GzB expression of CD3, CD8, CD4, NK, NKCD4 and NKCD8 cells from healthy control, lung cancer patients before, during and after induction of chemotherapy (after culture). **(B)** Levels of Intracellular GZB expression on CD3, CD8, CD4, NK, NKCD4 and NKCD8 cells from healthy control (white bars), lung cancer patients before, during and after induction of chemotherapy (black bars) after culture

The experiments above were done on fresh PBMCs. We then asked whether short activation of these cells in vitro can modulate the dysregulation in the phenotypes and cytolytic capability of these cells. To this end, we cultured fresh PBMCs from lung cancer patients and controls with 5 µg/ml Con A and 50ng/ml IL-2 for 3 days. After culture, the expression of GzB by cytotoxic CD8^+^ T, CD4^+^ helper T cells, NK, CD4^+^ NK T and CD8^+^ NK T cells were found to decrease significantly in lung cancer patients before and during induction of chemotherapy. Of note, patients after induction of chemotherapy showed the lowest levels of GzB as compared to the healthy controls **(**Fig. 4**).**

### Lower numbers of T, NK and CD56^+^T cells in PBMC from lung cancer patients after their activation

To understand whether the lower expression of GzB by in vitro activated PBMCs in lung cancer patients found above **(**Fig. 3**)** is due to alteration in the numbers of these cells in culture, we analyzed the numbers of each cell population after 3 days of culture. The fold change of cytotoxic CD8^+^ T, CD4^+^ helper T cells, NK, CD4^+^ NK T and CD8^+^ NK T cell populations greatly increased in patient before induction of chemotherapy when compared to healthy individuals. No changes were observed in the numbers of these cells in patients during and after induction of chemotherapy as compared to healthy individuals.

### Increased GzB expression in T and NK cells by in vitro activated PBMCs from lung cancer patients

**Fig. 5:**
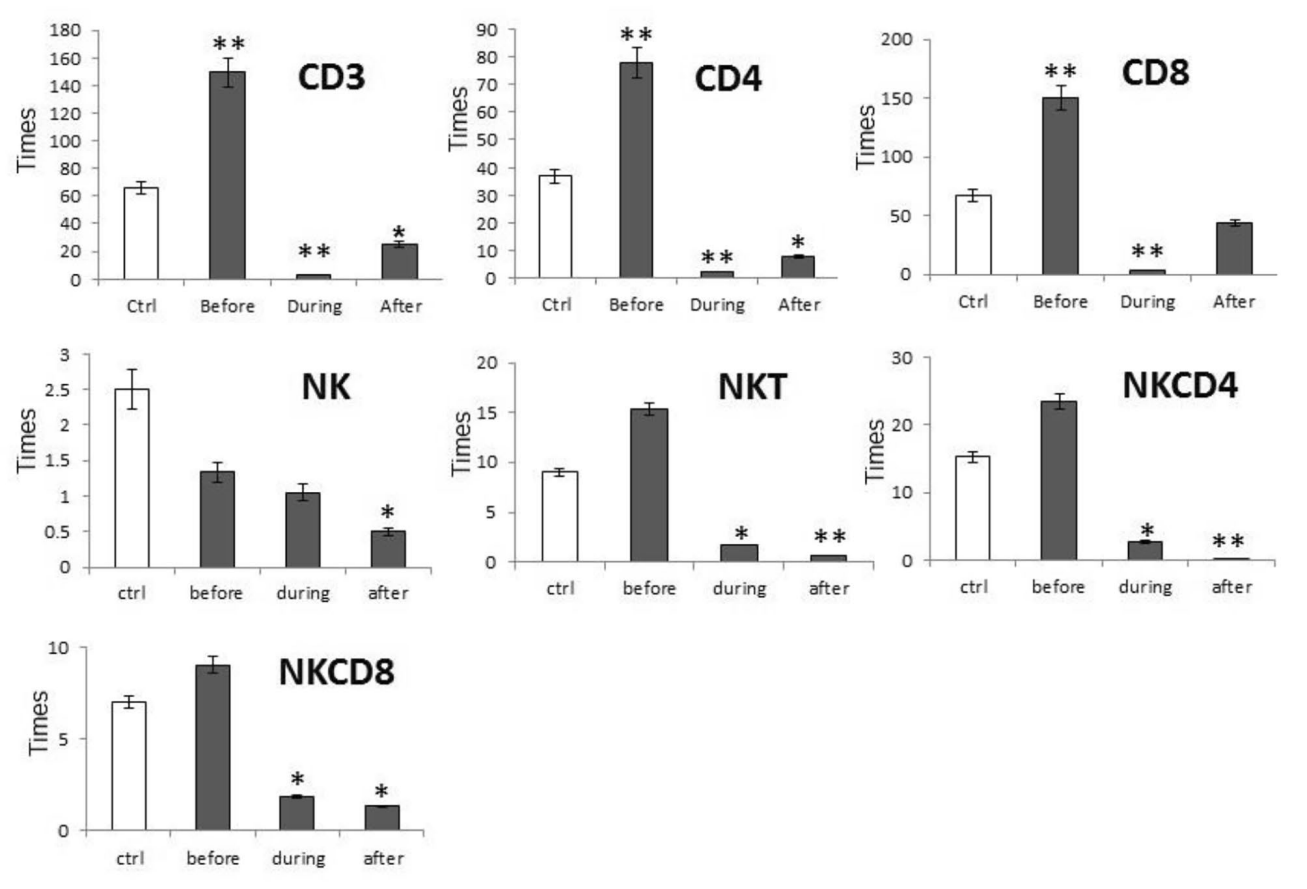
Statistical analysis of the fold change GZB expression from CD3, CD4, CD8, NK and NKT, NKCD4 and NKCD8 cells of lung cancer patients before, during and after induction of chemotherapy (black bars), compared to healthy control (white bars) in compare to samples after culture in the presence of Con A and IL-2. Data were analyzed using SPSS software and Mann–Whitney or one-way analysis of variance (a nova) with post-hoc for non-parametric analyses, P-values < 0•05 were considered significant (*and ** p ≤ 0.05, p ≤ 0.01)

To test whether the cytolytic activity of T and NK cells from lung cancer patients can be recovered after stimulation of these cells, we measured GzB expression by the T and NK cell subsets indicated above in Fig. 4. We found that patient before induction of chemotherapy showed increases in the numbers of GzB + T and NK cell subsets, including cytolytic CD8^+^ T, CD4^+^ helper T cells, CD4^+^ T and CD8^+^ T cells as compared to healthy individuals, while the numbers of GzB + NK cells decreased; all data are compared to healthy individuals. Patients during and after induction of chemotherapy showed significant decrease of cytotoxic CD8^+^ T, CD4^+^ helper T cells, NK, CD56^+^T, NKCD4^+^T and NKCD8^+^T cells when compared to healthy individuals (Fig. 5**).**

## Discussion

Cytotoxic T cells, NK and NK T cells are indispensable factors in the body’s ongoing defense against transformed tumor cells [23]. Several studies have reported that this local impairment of immune cells renders them less tumoricidal and thereby supporting the progression of cancer [24]. As increasing evidence suggests that tumors are able to regulate different immune arms at the levels of numbers and functions, we designed our study to clarify the changes in the peripheral immune environment in terms of numbers and functionality of immune cells present in peripheral blood of lung cancer patients under activation conditions [25]. In our system, we used Con-A, which is known for its effect as a stimulator of lymphocyte blastogenesis and mitosis, resulting in polyclonal activation of T cells [26, 27]. As of IL-2, it is considered as a key growth factor or activated T lymphocytes, resulting in massive expansion of T cells in vitro and in vivo [28–30]. Extensive studies on IL-2 have led to the clinical utilization of this molecule in patients with advanced cancer however this was limited due to the significant systemic side effects associated with its use. For this reason, attempting to overcome this obstacle without decreasing the antitumor potency current research is oriented towards developing combination regimens that have additive or synergistic antitumor effects with fewer toxicities to that activated CD8 T cells initially undergo IL-2-independent proliferation and its requirement for IL-2 as a growth factor gains prominence [31]. Our study demonstrated that the combined use of IL-2 and Con-A promoted synergistic *in-vitro* expansion and activation of peripheral immune cells, not only CD8^+^ T cells but also CD56^+^T cells have a major changes in their functionality and the power of secretion of the lysing granzyme which is not founded in the normal cells, this effect is notable in the functionality of the cells reflecting the effect on the cells. Therefore, the combined use of IL-2 and Con-A can be considered a practical strategy for the generation of antitumoral responses *in-vivo* and for future clinical applications.

Our study involved diagnosed patients with lung cancer at different stages compared to healthy donors. Our specific aim was to determine the functionality of total T cells and their subsets, including cytotoxic CD8^+^ T, CD4^+^ helper T cells, NK, CD56^+^T, NKCD4^+^T and NKCD8^+^T cells in lung cancer patients before, during and after induction of chemotherapy using a polyclonal activation approach consists of 5 µg/ml Con A and 50ng/ml IL-2. We used this approach to test our hypothesis that the peripheral immune components are affected by the tumor microenvironment ending with decreasing the number and function of immune cells and whether this impact can be recovered after activation. We found decreases in the numbers and functions of the studied cells in fresh blood of cancer patients in particular after chemotherapy. Interestingly, however, this dysregulation in immune cells was recovered after cell activation.

Our results with regard to the decreases in the numbers and function of the studied CD4 + and CD8 + T cells as well as NK cells are in line with results from a previous study which concluded that the numbers of circulating T, NK and CD56^+^T cells and their levels of GzB expression are decreased in lung cancer patients. This as our previous study could not explain the mechanisms behind this dysregulation in immune cell numbers and the cytolytic function [32]. This dysregulation, however, could be attributed at least in part by dysregulation in the cytokine/chemokine microenvironment that can shape the overall immune responses of T and NK cells.

Circulating cytokines are closely associated with the immune status against several diseases, in particular cancer [33]. In the present study, we analyzed the levels of inflammatory and pro-inflammatory cytokines/chemokine axis in different groups of patients as compared to individuals controls. As shown in our previous study, the levels of IL-1 and CXCL8 in early diagnosis patients showed 37- and 40-fold increases, respectively as compared with the control group. These cytokines have been reported to promote tumor growth, chemo-resistance and help in sustaining an immunosuppressive milieu in both lung and breast cancer cells is an important regulatory mechanism for cell growth and invasion [34, 35]. Additionally, we found that the levels of IL-6 were increased by 58-fold in lung cancer patients, which is also in line with previous studies that has reported a positive correlation of the increased levels of this cytokine and lymph node metastasis, distant metastasis and worse overall survival. IL-6 was found to mediated inflammation in NSCLC-related morbidity and mortality [34, 36, 37]. This would explain the efficacy of Lin et al. supports that with targeting IL-6 signaling pathway as an important strategy for treating lung cancer [38]. Furthermore, CXCL8 which promotes angiogenesis and tumor progression was increased by 40-fold in lung cancer patients as compared to healthy controls. This is consistent with Debnath et al. who showed that the increased serum levels of CXCL8 in patients with prostate cancer as compared to healthy volunteers [39]. The levels of CXCL10, IL-5, CCL11 and CCL4 showed no significant difference between our lung cancer patients and the control group [40]. Taken together, the present study supports the hypothesis that systemic cytokine cascade exists in lung cancer patients, reflecting the tumor stage and host immune status. These changes may be of a high importance as a diagnostic and prognostic tool in lung cancer as well as potential targets for immunotherapy.

By analyzing the cytolytic capacity of T and NK cell subsets in the present study, we found decreases in the levels of GzB by these cells from patients before and during induction of chemotherapy when compared to CTRL. Interestingly, however, we observed increases in GzB secretion by these cells in patients after induction of chemotherapy. This combined deficiency in the number and functionality of various lymphocyte subsets can be explained by the impact of soluble mediators such as PGE2, TGF-β produced from the tumor cells, consisted with Zhao et al., who that these as suggested before that these mediators are responsible for inhibition of CTL and NK function [16, 41]. On the other hand, these findings may also be attributed to the effect of immune suppressive cells such as myeloid-derived suppressor cells (MDSCs) and regulatory T cells (Treg), which we and others have reported to be correlated with the stages and cancer progression excreting their effect on immune cells [42, 43].

Interestingly, after cultivation of PBMCs in vitro, changes were observed in most populations of cells, showing increases in the numbers of cells in patients before, during and after chemotherapy. The increases in the cell numbers was more pronounced in patients before induction of chemotherapy, in particular for total T cells, cytotoxic CD8^+^ T, and NKCD4^+^T cells in specific [32]. Similarly, the fold change in the expression of GzB on total T cells, cytotoxic CD8^+^ T, CD4^+^ helper T cells, CD56^+^T, NKCD4^+^T and NKCD8^+^T cells after culture were also significantly increased in patients before induction of chemotherapy when compared to individual controls and other lung cancer patients. Of note, we also found that CD4 + T cells can secrete significant amount of GzB which is almost similar in quantity to those of CD8 + T cells which is may the effect of Con-A which is known as a stimulator of lymphocyte blastogenesis and mitosis [44].

Con-A have important role in inducing T lymphocytes of BALB/c mice as polyclonal activators [26, 27]. also, IL-2 has been considered to be a key growth for antigen-activated T lymphocytes since it also maintains self-tolerance and causes a massive expansion of CD8^+^ T cells and regulate distinct aspects of primary T-cell expansion in vivo [28–30]. Extensive studies on IL-2 have led to the clinical utilization of this molecule in patients with advanced cancer however this was limited due to the significant systemic side effects associated with its use. For this reason, attempting to overcome this obstacle without decreasing the antitumor potency current research is oriented towards developing combination regimens that have additive or synergistic antitumor effects with fewer toxicities as reported by Lefrançois to that activated CD8 T cells initially undergo IL-2-independent proliferation and its requirement for IL-2 as a growth factor gains prominence [31]. Our study demonstrated that the combined use of IL-2 and Con-A promoted synergistic *in-vitro* expansion and activation of peripheral immune cells, specifically CD8^+^ Tcells, this effect is notable in the functionality of the cells reflecting the effect on the cells Therefore, the combined use of IL-2 and Con-A should be considered a practical strategy for the generation of antitumoral responses *in-vivo* and for future clinical applications.

On the contrary, the NK population of cells was not affected by the stimulatory effect by Con A and IL-2 in terms of either numbers or functionality. This may suggest that NK cells are not activated in vitro as compared to cytotoxic CD8 + T cells. Meanwhile, we found that NK cells showed high production levels of IFN-γ (data not shown) and reported in [45, 46], explaining why the expression levels of GzB by NK cells were not potently affected, These data also support the notion that CTL are the main secretors of GzB.

In our system, we found that CD56^+^T profile showed slightly increase in numbers without significance effect on GzB expression. This low cytolytic function of NK cells can be attributed to the release of soluble major histocompatibility complex (MHC) class I chain-related molecules by cancer cells, resulting in impairing the lytic activity via down-regulation of the NKG2D receptor [47].

## Conclusion

The results of this study confirm that the functionality of CTL and NK cells are impaired in cancer patients during and after induction of chemotherapy, while, patients before induction of chemotherapy may have the ability to improve their CTL functionality under activation conditions such as combination of Con-A and IL-2.

## Data Availability

The data that support the findings of this study are available when requested from the corresponding author, [M.L.Salem], upon reasonable request.
